# Effectiveness and impact of community-based health insurance on health service utilization in northwest Ethiopia: a quasi-experimental evaluation

**DOI:** 10.3389/fpubh.2023.1078462

**Published:** 2023-11-13

**Authors:** Samrawit Mihret Fetene, Mezgebu Yitayal Mengistu, Andualem Yalew Aschalew

**Affiliations:** Department of Health Systems and Policy, Institute of Public Health, College of Medicine and Health Sciences, University of Gondar, Gondar, Ethiopia

**Keywords:** evaluation, outcome evaluation, community-based health insurance, health service utilization, effectiveness, Ethiopia

## Abstract

**Background:**

Addressing the health challenges of lower socioeconomic groups in Ethiopia is still a huge problem. In that regard, the government piloted the community-based health insurance (CBHI) in 2011 in a few districts and subsequently scaled up. However, the effectiveness of the program on the utilization of health services and its impact was not well explored. Thus, we aimed to evaluate the effectiveness of CBHI toward health services’ utilization and its impact in northwest Ethiopia.

**Methods:**

A quasi-experimental matched comparison group evaluation design with sequential explanatory mixed methods was employed. To evaluate the CBHI program, the effectiveness and impact dimensions from the Organization for Economic Cooperation and Development framework were used. A multistage sampling technique was used to select a total of 332 households enrolled in the CBHI program; 341 comparison households who did not enroll in the program were also randomly selected. A structured interviewer-administrated questionnaire was used to evaluate the effectiveness and impact of CBHI on health service utilization. The Propensity score matching model was employed for the estimation of the effect of the CBHI program on health service utilization. Challenges for program achievement toward health service utilization were explained through qualitative data and these were then analyzed thematically.

**Results:**

The evaluation showed 1.3 visits *per capita* per year of health service utilization among CBHI members. Households enrolled in CBHI increased health service utilization by 6.9 percentage points (ATT = 0.069; 95% CI: 0.034, 0.114). There was an improvement in health service utilization after the introduction of CBHI, however, there are challenges: (i) shortage of human resources, (ii) out-of-stock of drugs and medical supplies, and (iii) long waiting times for service and reimbursement claims. These issues limit the success of the program toward health service utilization.

**Conclusion:**

The CBHI program contributed to health service utilization improvement among CBHI members. However, the utilization rate of health services among CBHI members is still less than the target stated for the program and also the WHO recommendation. Therefore, the findings of this evaluation can be used by program implementers, policy makers, and other stakeholders to overcome the identified challenges and to increase the success of the program.

## Introduction

Impoverished households often face significant financial barriers due to out-of-pocket (OOP) medical expenditures (1). Globally, around 150 million people experience financial catastrophes and 100 million people have experienced impoverishment due to Out-of-pocket expenditure every year (2). The high reliance on out-of-pocket financing causes individuals to reduce health-care utilization, thereby prolonging or worsening health, and putting them at risk of destitution (3–5). OOP expenditure is the most inequitable way of financing and leads to health-related impoverishment, predominantly for the poor in developing nations (6).

Moving from OOP to pre-payment is an important step toward averting financial hardship. In that regard, the community-based health insurance (CBHI) is an emerging alternative to increase primary healthcare access through the reduction of financial barriers (7). The program was advocated as a transitional mechanism to achieving universal coverage for health in low-income countries, especially for people living rurally due to their inability to access quality health care services provided by their respective government (8). All types of essential health services that would be covered through out-of-pocket spending are covered by the CBHI program (9). The Ethiopian government endorsed and launched CBHI in 13 pilot districts in Tigray, Amhara, Oromia, and the Southern Nation and Nationality of People of Ethiopia region in 2011 that aimed to provide risk protection by alleviating financial constraints and lowering OOP health care expenditure and ultimately to attain universal health coverage (UHC) (10, 11). Differences between scheme members and non-members in utilization of modern health care were reported by a number of studies (12–21). For instance, a study done in Bangladesh revealed that health service utilization was significantly higher in CBHI members (50.7%) compared to non-members (39.4%) (17). Similarly, a study conducted in India indicates that there were statistically significant differences in the average number of health-care visits between insured and uninsured people (15). In Burkina Faso, a study showed that the outpatient visits among members were about 40% higher as compared to non-members, while the differential effect on utilization of inpatient care between insured and non-insured groups was insignificant (13).

There have been several studies conducted in Ethiopia on different issues related to the CBHI program: initial enrollment status (22–25), drop-out rate (26–29), membership renewal status (30, 31), and satisfaction (32–34). Some literature also revealed the impact of CBHI on utilization of health-care services in Ethiopia (35–37). In one study from North-West Ethiopia, the health service utilization rate for CBHI scheme members was 50.5%, while for non-members it was 29.3% (38). Following implementation, the ministry of health performed an evaluation of CBHI in these pilot areas for scaling up and found that 72.3% of members visited health facilities while 69.3% of non-members did the same (35). In addition to this, a study on the impact of Ethiopia’s pilot CBHI scheme on health service utilization found that utilization of outpatient services were 35 and 22% for CBHI members and non-members, respectively, (39). However, very few of these studies supplemented their quantitative findings with qualitative data.

While the CBHI program has expanded, the government still has great concerns about the sustainability of the CBHI scheme. In particular, they question whether CBHI scheme enrollment has increased CBHI member health service utilization in different contexts. Further, there has been research regarding the role of CBHI on health-care utilization among enrolled members compared to non-members in Ethiopia in general (36) and northwest Ethiopia in particular (37, 38, 40). Prior studies have been pure research, whereas our study is an evaluation that allows us to make value judgments about the impact of the program on health service utilization. Studies in the past have focused on specific health services, including outpatient health services (41, 42), maternal health service (43), and childhood services (44). In addition, health service utilization was assessed during illness episodes, meaning they measured only therapeutic service utilization (37, 45), while in our study, we examined all types of service utilization, including diagnostic, therapeutic, and follow-up services.

In most cases, social interventions such as CBHI are based on observational studies. These studies may introduce several types of biases when comparing outcomes between those who receive social intervention and those who do not. Studies of this type may not have comparable subjects before the intervention, so differences in outcomes may not reflect the true effects of the intervention (13). In recent times, propensity score matching (PSM) has been employed to observe various impacts of health insurance (15, 46–48). In this study, we use primary data to compare “treated” and “untreated” individuals in areas where CBHI has been implemented and not implemented, respectively. Moreover, using PSM in the analysis helps to decrease bias from observables. The qualitative component of our study also contributed to the interpretation and validation of our findings.

Therefore, this outcome evaluation aimed to evaluate the effectiveness of CBHI and its impact on health service utilization in northwest Ethiopia. Based on the evidence, strategies can be developed to improve the effectiveness of the program. If the government follows through on the findings, it will ensure the sustainability of the program in the future. Moreover, it will be incredibly helpful to other researchers wishing to conduct similar research in the future.

### Overview of CBHI program (intervention)

CBHI implementation began in 2010/11 as pilot schemes in 13 Woreda of Amhara, Oromia, Southern Nation and Nationality of People of Ethiopia region (SNNP), and Tigray regional states. The benefit package includes both outpatient and inpatient service utilization at public facilities but excludes treatment abroad and treatments with large cosmetic value such as artificial teeth and plastic surgery. The referral procedure requires members to visit health centers before they can be referred to hospitals; those who do not follow this referral procedure need to cover half the costs of their medical treatment (42).

The CBHI program carries out four main responsibilities: (i) identifying and signing contracts with health facilities to provide care to program members, (ii) reimbursing providers for the health care utilized by beneficiaries, (iii) financial administration, and (iv)database management including membership, premium payment, and utilization of health care by members (49).

Currently, 1920 health centers and 245 hospitals are providing health services to CBHI beneficiaries through contractual agreements with the CBHI program. Various initiatives were implemented to ensure provision of quality health services to beneficiaries. These include development of a CBHI clinical audit manual, provision of training to CBHI staff, undertaking of clinical audits, and assessment of drug availability and facility readiness on CBHI contracted health facilities. Moreover, quality improvement platforms involving health sector stakeholders were carried out (35). Currently, the program is scaled up into different Woreda and the program is in the implementation stage (43).

### Stakeholder identification and analysis

The evaluability assessment was conducted from 1 to 30 December 2020 using a checklist on program design, information availability, practicality, and utility. The overall score was 72.8%, so the evaluation was able to proceed (If the evaluability assessment ‘Yes” is greater than 50, it is able to be evaluated). Stakeholder analysis was also performed using a participatory approach to identify the program implementer. Health facilities contracted with the program and those who had an interest in the program were engaged, i.e., Amhara regional health office, Amhara regional CBHI office, Woreta town CBHI office, Woreta health office, CBHI contracted health institution managers, health workers in CBHI contracted health facilities, and members.

**Figure 1:**
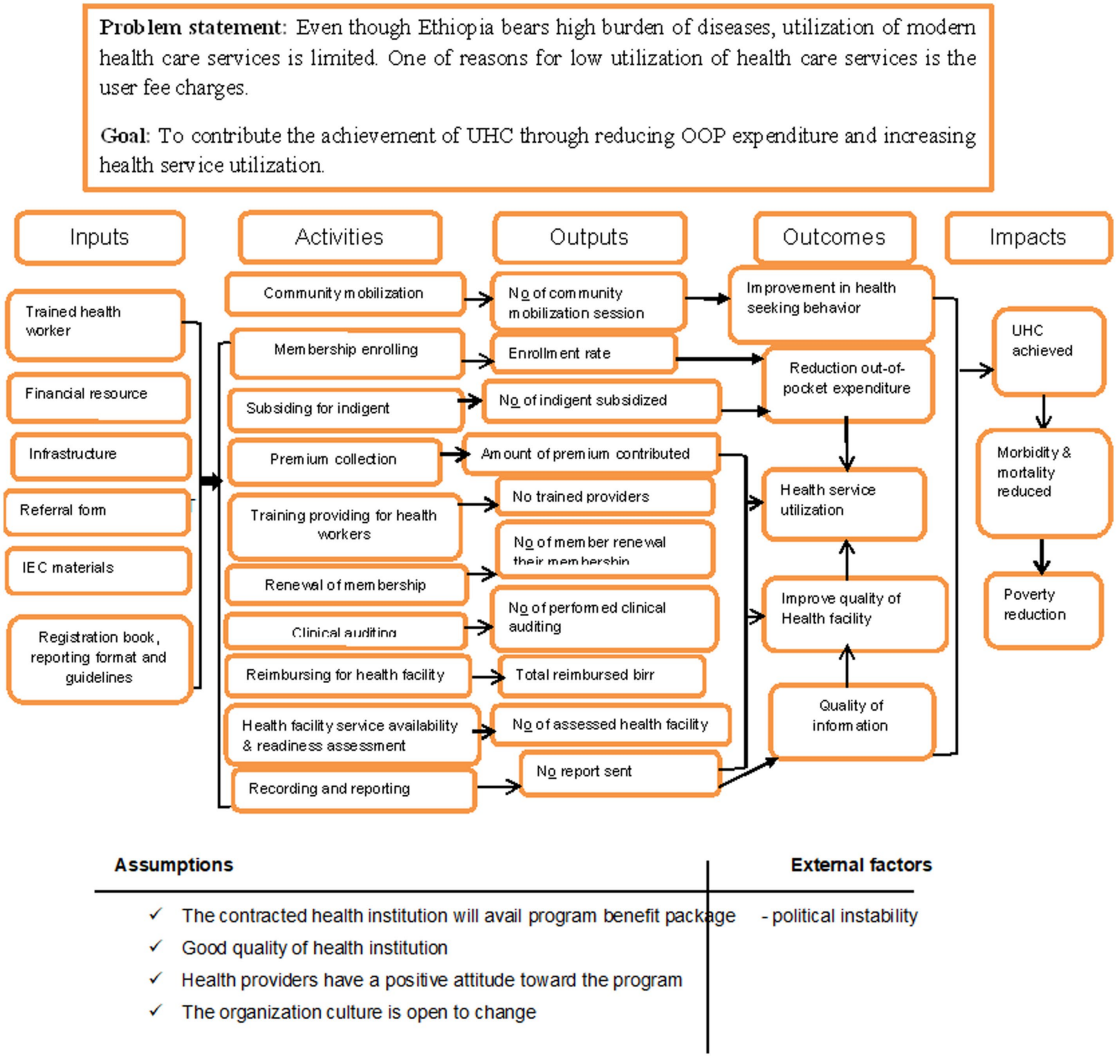
The logic model of CBHI program Woreta Town, Ethiopia, 2021.

The stakeholders were communicated with through cellphone and in-person communication. CBHI stakeholders were identified and their role in the program and evaluation was discussed. They were able to define and identify the priorities of the program and raise their concerns. Finally, they agreed on the evaluation questions and dimensions as well as the judgment criteria. Figure 1 shows the logic model of the Community Based Health Insurance Program, Ethiopia, 2021.

## Materials and methods

### Evaluation design and contexts

We used a matched comparison group evaluation design with sequential explanatory mixed methods to evaluate the outcome of the CBHI program on health service utilization. The groups were towns that had implemented already CBHI (Woreta) and those that had not yet started (Dabat). They were matched based on socio-demographic, health-related factors, and health facility-related factors.

Woreta towns where CBHI was implemented (intervention group) and Dabat towns where CBHI had not yet started (comparison group). Woreta town, 625 km from Addis Ababa, is located in the South Gondar zone of the Amhara National Regional State. According to the 2017 population projection, the town has an estimated total population of 36,244, of whom 16, 954 are males. Currently, there is one health center providing health services for the community. The CBHI program was implemented in 2017 and, currently, around 3,809 households are members of the program. Dabat town is located 821 km from Addis Ababa in the north Gondar zone of the Amhara National Regional State. According to the 2017 population projection, the town has a total population of 27,510, of whom 12,759 are males. Currently, there is one health center providing health services for the community.

### Source and study population

The source and study population for the intervention group and the comparison group were household heads who were CBHI members in Woreta town and household heads who worked in the informal sector in Dabat town.

Household heads who had been a member of the CBHI program for 1 year or longer among the intervention group with a renewed CBHI card at the time of interview were included in the study. For the comparison group, household heads who lived in Dabat for 1 year or longer were included in the evaluation study. Household heads who visited exempted services were excluded from both groups, and household heads who were government employees from the comparison group were also excluded from the study.

For the qualitative study, the Amhara regional CBHI coordinator, district CBHI officer, CBHI contracted health facility manager, and CBHI members were the study participants.

### Evaluation approach, focus, and dimensions

In this evaluation, a formative approach that focused on the outcome of CBHI was used (Figure 1). The Organization for Economic Cooperation and Development (OECD) framework was used to select the evaluation dimensions (50). It has five dimensions: relevance, effectiveness, efficiency, impact, and sustainability. Accordingly, effectiveness and impact dimensions were customized based on the interest of key stakeholders, their questions, and the feasibility of the evaluation.

### Sample size and sampling procedures

#### Quantitative data

The sample size was determined using a power approach (double population formula) from a previous study (51) by considering power 80%, confidence interval 95%, proportion of health service utilization among CBHI non-member households 20%, proportion of health service utilization among CBHI member households 35%, a ratio of 1:1, a design effect of 2, and non-response 10%. Therefore, the final sample size was 708 (354 for intervention and 354 for non-intervention).

A multistage sampling technique was used to select kebeles and households in each town. In Woreta town, two kebeles out of the total four kebeles were selected randomly using the lottery method. Similarly, three kebeles out of the total five kebeles were selected randomly in Dabat town. Then households were selected systematically every sixth interval from each of the selected kebeles. The list of CBHI program members and informal sector workers were obtained from each kebele administration office.

#### Qualitative data

A qualitative study was conducted in Woreta town, where the CBHI program is implemented. Qualitative data were collected to examine why the program cannot meet the demand for health service utilization as well as to explore the extent of supply side factors affecting CBHI program effectiveness.

A purposive sampling procedure was used to select participants for in-depth and key informant interviews. The Amhara regional CBHI coordinator, district CBHI office, and CBHI contracted health facility manager were selected for key informant interviews and CBHI members were chosen for in-depth interviews.

As a result, one regional CBHI coordinator, one district CBHI officer, and one CBHI contracted health facility manager were included in the key informant interviews. We reached saturation with the eighth in-depth interview with CBHI members.

### Definition and measurements of variables

Health service utilization in our evaluation refers to household members who visited health facilities in the previous 12 months prior to the evaluation. Health facility visits included visits for diagnostic, follow-up, and treatment services. It was a dichotomous variable based on the survey question “Did one of your family members visit the health facility in the previous 12 months? (38). According to WHO recommendations, we categorized the health service utilization as “utilized” when at least one household visited a health facility two or more times in the previous 12 months. Otherwise, it was classed as “not utilized” (52).

Effectiveness refers to what extent the objectives of the CBHI program toward health service utilization were achieved (50). This dimension was measured using the revised health management information system indicator: current *per capita* health service utilization for CBHI beneficiaries (53). Current *per capita* health service utilization of CBHI beneficiaries was then compared against the CBHI program stated objective: 1.6 visits *per capita* per year (54).

*Per capita* health service utilization is the number of visits that each CBHI household member made to a health facility within a year divided by the number of each household members with CBHI in the town (53). It was calculated using the formula


$$\frac{\begin{array}{c} \mathrm{Total}\;\mathrm{number}\;\mathrm{of}\;\mathrm{visits}\;\mathrm{made}\;by\;\mathrm{CBHI}\;\mathrm{members}\\ to\;\mathrm{health}\;\mathrm{facilities}\;\mathrm{within}\;a\;\mathrm{year} \end{array}}{\begin{array}{c} \mathrm{Total}\;\mathrm{number}\;\mathrm{of}\;\mathrm{CBHI}\;\mathrm{members}\\ \mathrm{within}\;\mathrm{selected}\;\mathrm{households} \end{array}}$$

Impact was measured by calculating the net effect of the CBHI program on health service utilization. The treatment and comparison groups are typically identified in matched comparison group designs after the program has already been implemented (55). Accordingly, the town under the CBHI program, i.e., Woreta town, was considered as an intervention group, and the town that was not under the CBHI program, i.e., Dabat town, was a comparison group. Thus, the design allows us to estimate the impact of the CBHI program on health service utilization in the intervention area. The average treatment effect was estimated using a propensity score method (PSM) by taking socio-demographic factors, health-related factors, and health facility-related factors to estimate the propensity score. The matching based on the propensity score was done using the Kernel matching method. Finally, the average treatment effect was estimated by the mean difference between the two statistically created groups.

Perceived health status: The participants reported about their health status (self-rated) and were assigned numerical values according to the following scale: 5-very good, 4-good, 3-medium, 2-poor, and 1-very poor. Then the values were re-categorized into good, medium, and poor (38).

Wealth index: We adapted urban dwellers’ questions from the Ethiopian Demographic and Health Survey (56) and asked participants 29 questions. The collected information included ownership of assets (e.g., car, refrigerator, and television) and housing characteristics (e.g., material of dwelling floor and roof and toilet facilities). All categorical variable and continuous variables were categorized between “0” and “1.” Then a principal component analysis was used to reduce the items. Further, all eligible factor scores were computed using the regression-based method to generate one variable: wealth status. Following this, the final scores were ranked to five quintiles as first, second, third, fourth, and fifth. Finally, ranks were coded as richest, rich, middle, poorer, and poorest, respectively.

The independent variables of the study were categorized into socio-demographic variables (household head’s age, sex, religion, wealth index, education, occupation, marital status, and family size); health-related variables (presence of chronic illness in the household, perceived health status of a household, household having less than five years, household having greater than 55 years, and illness history in the family); and health facility-related factors **(**distance from a health facility).

### Data collection tools and procedures

A structured interviewer-administered questionnaire was used to collect the necessary information about the CBHI program effectiveness and the impact of CBHI on health service utilization. An interview guide was used for the key informants and in-depth interviews.

The questionnaire was developed in English by reviewing different literature (14, 15, 21, 38, 57–59) and then translated into the local language (Amharic) and back-translated to English to maintain its consistency. Ten BSc degree graduate nurses and two public health professionals were recruited for quantitative data collection and supervision, respectively. Qualitative data was collected by the principal evaluator.

In the first phase, quantitative data were collected and analyzed to measure program effectiveness and its impact on service utilization. Qualitative data were collected in the second phase in order to explain the reason for the observed level of effectiveness. The interviews were held in a private setting and each interview lasted an average 40 min. Field notes were taken during the key informant interviews and in-depth interviews.

### Data quality assurance

To assure the quality of quantitative data, one-day training was given on the evaluation objectives, data collection tools, and basic techniques of data collection and supervision. In addition, a pretest was conducted on 5% of the sample (34 households) outside of the study area (Bahir Dar city). Based on the pretest findings, the final versions of the tools were modified before the actual data collection. Supervisors and principal evaluators checked the data accuracy, consistency, and completeness on a daily basis.

Interviewees were asked whether the findings were consistent with their own experiences in order to ensure the trustworthiness of qualitative data. A peer review was performed on the second draft of the transcript, codes, and concepts by several colleagues. Documentation was done throughout the study to ensure the conformability of the findings. Moreover, the purposive sampling method and experienced professionals were used to validate the findings’ dependability and transferability (60).

### Data management and analysis

The quantitative data were entered using Epi Data version 4.6 Software and then exported to STATA version-14 for cleaning, coding, and further analysis. Descriptive statistics were used to calculate frequency, percentage, mean, and standard deviation of both outcome variable and the independent variables. The quantitative data were analyzed first. A propensity score matching (PSM) approach was used to estimate the net effect of the CBHI program on health service utilization in order to minimize the baseline differences in the characteristics. Propensity score matching analysis is a causal inference technique for treatment effect estimation in observational studies by accounting for the conditional/ probability of treatment selection (61).

### Covariate selection

Household head age, educational status, sex, marital status, family size, religion, occupation status, wealth index, family member chronic illness status, distance to the nearest health facility, family member younger than 5 years, family member older than 55 years, illness history in past 12 months, and perception about family health status were measured and included in the analysis. Thus, covariates were identified by considering different theories and extant empirical evidence to conceptualize covariates causally related to exposure and outcome (62). This included covariates that occurred before the program and covariates that are highly associated with the outcome (63) as well as covariates that affect the membership of the program and the outcomes (64). The measurement reliability of covariates was also considered because unreliably measured covariates may not only fail to reduce bias in estimated treatment effects but may indeed add bias (65).

### Estimating propensity score

**Figure 2:**
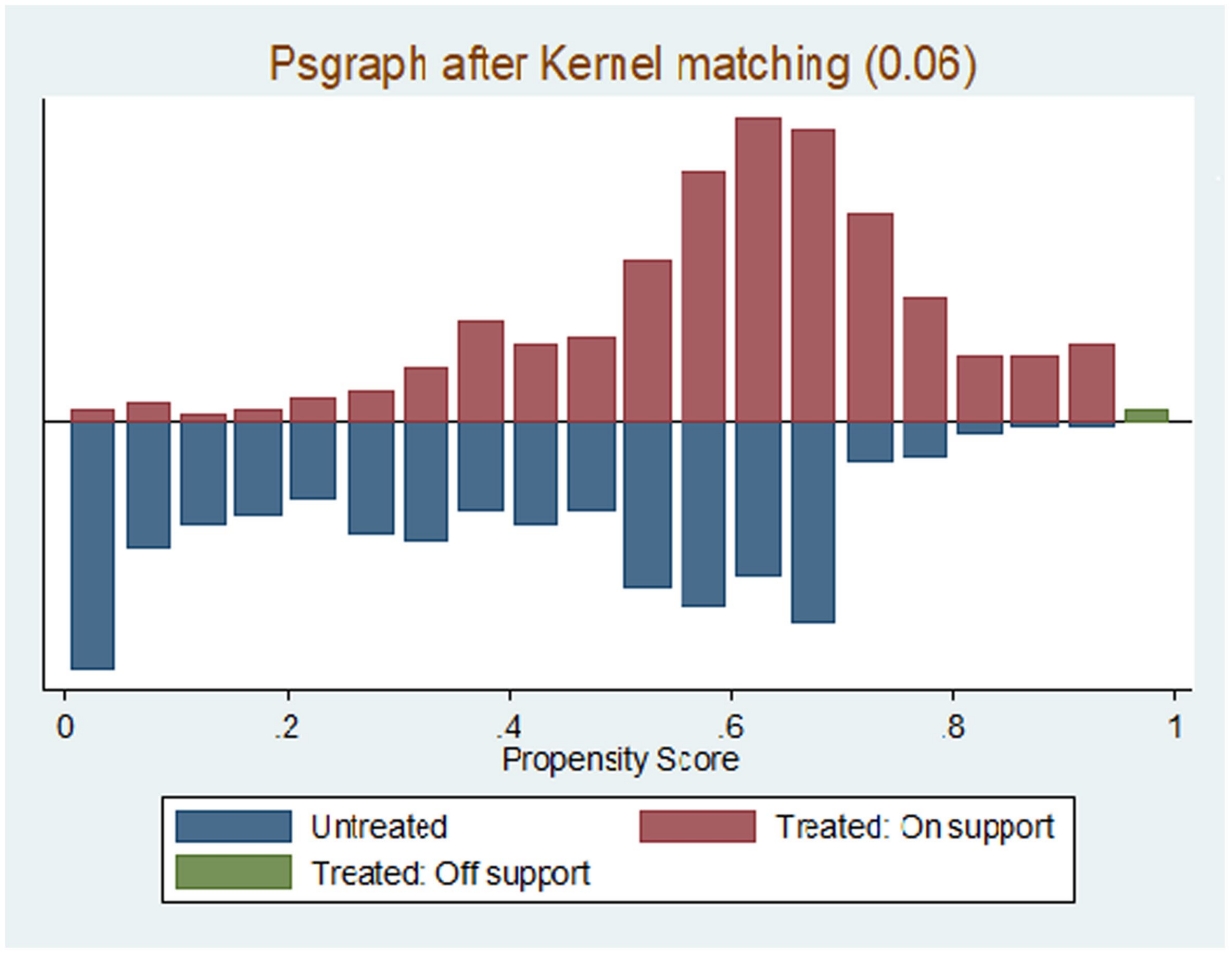
Psgraph after kernel matching (0.06).

The propensity score was estimated using probit regression with the identified covariates. The propensity matching property was satisfied, which means the propensity score had common support (Figure 2).

### Selection and performance-checking of matching algorithm

The different matching algorithms including nearest neighbor matching (NNM) with and without replacement, NNM with the caliper, radius matching, and kernel matching algorithm were employed and checked. However, there is no specific matching algorithm that wins for a particular evaluation (66). Pstest was conducted to see the distribution of the covariates between the intervention group and comparison group before and after matching as well to select the matching algorithm that gave the highest-quality match between the two groups.

The Pstest provides the standardized bias, mean bias, pseudo-*R*^2^, likelihood ratio test, t-test results, and variance ratio (67). Kernel matching algorithm (bandwidth 0.06) was selected because it gives the lowest mean bias 2.5 (before matching mean 20), post-matching standardized bias across all covariates less than 5% (before matching 11 covariates standardized bias was greater than 10%), insignificant *t*-test (no difference between two groups), and variance ratio for a continuous variable of 0.62. Pseudo-R squared values were also compared before and after matching, which indicates how well the covariates explain participation in the program. The low pseudo-R^2^ and insignificant likelihood ratio tests supported the hypothesis that both groups had the same distribution in covariates after kernel matching with bandwidth 0.06. Therefore, matching quality is good after kernel matching with bandwidth (0.06). As a result, kernel matching was selected to estimate average treatment effect.

Finally, audio records were transcribed in the local language and translated to English. The qualitative data were analyzed using thematic analysis. Though the result is dominantly quantitative, the qualitative data were used to complement the quantitative findings and we presented the qualitative data under the quantitative finding.

### Judgment matrix

The judgment criterion for health service utilization was adopted from different evaluation studies on the CBHI program (13, 15, 17). The effectiveness judgment criteria were prepared and agreed upon with stakeholders (Table 1).

**Table 1:** The judgment matrix of CBHI outcome in Dabat and Woreta towns, northwest Ethiopia, 2021.

Dimensions	Score	Judgment
Effectiveness	Visit *per capita* ≥1.6 = Agreed Effectiveness= $\frac{\mathrm{Visit}\;\mathrm{per}\;\mathrm{capita}\;\mathrm{per}\;\mathrm{year}\;}{1.6}$ *100	>90% = Excellent
		80–90% = Very Good
		70–80% = Good
		60–70% = Fair
		<60% = Poor
Impact	Average treatment effect on treated (68) ≥10 = Agreed Impact= $\frac{ATT}{10}$ *100	>90% = Excellent
		80–90% = Very Good
		70–80% = Good
		60–70% = Fair
		<60% = Poor
	$\frac{\left(\;\mathrm{Effectiveness}+\mathrm{impact}\right)}{2}$	>90% = Highly satisfactory
Overall outcome		75–90% = Satisfactory
		50–75% = Unsatisfactory
		<50% = Highly unsatisfactory

### Ethical considerations

Ethical approval was obtained from the Ethical committee of the Institute of Public Health, College of Medicine and Health Sciences, University of Gondar (Ref No: IPH/1461/2013). All study participants were oriented on the objectives and purpose of the study before study participation. Participants were informed that participation had no impact on membership status. Written informed consent was obtained, and study team members safeguarded the confidentiality and anonymity of study participants throughout the entire study. In order to protect the identities of the study participants, each participant was given a unique identification number. Participation in the study was voluntary, and individuals were free to withdraw or stop the interview at any time. This study was conducted in accordance with the Declaration of Helsinki.

## Results

### Characteristics of the study participants

Table 2 describes the socio-demographic and economic characteristics of the study participants. A total of 332 and 341 households from CBHI members (intervention group) and non-CBHI members (comparison groups) were interviewed with a response of 93.8% and 96.2, respectively. Of the respondents, 63.25 and 53.67% of the study participants were males from the intervention and comparison group, respectively. Nearly 49.10 and 35.18% of the participants were daily labor workers in the intervention and comparison group, respectively. More than half (53.01%) and 50.15% of the study participant’s family size was greater or equal to five among the intervention and comparison group, respectively. In the intervention group, 40.36% of household heads were not able to read and write, whereas this amount was 56.01% in the comparison group. Moreover, 27.41% of participants in the intervention group and 25.22% in the comparison group were in the middle household wealth index.

**Table 2:** Socio-demographic characteristics of participants in Dabat and Woreta town, northwest Ethiopia, 2021 (*n* = 673).

Variables	CBHI members		CBHI non-members	
	Frequency	Percentage (%)	Frequency	Percentage (%)
Age in years				
18–24	1	0.30	20	5.87
25–44	169	50.90	141	41.34
45–65	146	43.98	119	34.90
>65	16	4.82	61	17.89
Sex of respondent				
Male	210	63.25	183	53.67
Female	122	36.75	158	46.33
Educational status				
Unable to read and write	134	40.36	191	56.01
Able to read and write	96	28.92	29	8.50
Primary school	43	12.95	59	17.30
Secondary school	51	15.36	47	13.78
Other*	8	2.41	15	4.40
Family size				
1–4	156	46.99	170	49.85
≥ 5	176	53.01	171	50.15
Religion				
Christian	306	92.17	337	98.83
Muslim	26	7.83	4	1.17
Marital status				
Married	227	68.37	231	67.74
Single	18	5.42	25	7.33
Divorced	39	11.75	20	5.87
Widowed	48	14.46	65	19.06
Household wealth index				
Poorest	72	21.69	67	19.65
Poor	61	18.31	82	24.05
Middle	91	27.45	86	25.22
Rich	33	9.94	47	13.78
Richest	75	22.59	59	17.30
Occupational status				
Farmer	19	5.72	15	4.40
Housewife	33	9.94	101	29.62
Daily labor worker	163	49.10	120	35.18
Merchant	104	31.32	48	14.08
Other**	13	3.92	57	16.72

*Diploma, Degree, **Private sector employee, sexual worker.

All the in-depth interview participants fell between the ages of 27 and 68 years. In terms of their educational backgrounds, they ranged from illiterate to having attended secondary school. The occupational status of participants included housewife, daily labor worker, farmer, and merchant.

### Health facility related characteristics

Nearly 98% of the households among the intervention group and 73.90% of households in the comparison group resided within five kilometers from the nearest health facility.

### Health-related characteristics

The health-related characteristics of study participants are presented in Table 3. More than three-fourths of households (98.48%) in the intervention group and 79.18% of households in the comparison group had at least one household member visit a health facility in the past 12 months. Ninety-nine percent of the households in the intervention and 99.71% in the comparison group reported that there was a history of illness in their household in the past 12 months. Nearly 71% of the intervention group perceived their household health status as good; 57.48% in the comparison group did the same.

**Table 3:** Health-related characteristics of the study participants in Dabat and Woreta town, northwest Ethiopia, 2021 (*n* = 673).

Variables	CBHI members		CBHI non-members	
	Frequency	Percentage (%)	Frequency	Percentage (%)
Family member ill in the past 12 months				
Yes	330	99.4	340	99.71
No	2	0.60	1	0.29
Has a sick person in the household visited a health facility in the previous 12 months?				
Yes	325	98.48	270	79.41
No	5	1.53	70	20.59
Perception about the health status of your family members (Self-rate)				
Poor	28	8.43	36	10.56
Medium	68	20.48	109	31.96
Good	236	71.08	196	57.48
Household member(s) younger than 5 years				
Yes	187	56.33	177	51.91
No	145	43.67	164	48.09
Household member(s) older than 55 years				
Yes	92	27.71	147	43.11
No	240	72.29	194	56.89
Anyone from the household with chronic illness				
Yes	88	26.51	63	18.48
No	244	73.49	278	81.52

### Effectiveness of CBHI on health service utilization

The total number of CBHI members who visited the health facility were 2023 and the total number of beneficiaries within the selected households were 1,545; that resulted in 1.3 visits *per capita* per year. The program has very good (81.3%) performance on the effectiveness dimension based on the judgment criteria.

### Impact of CBHI on health service utilization

**Figure 3:**
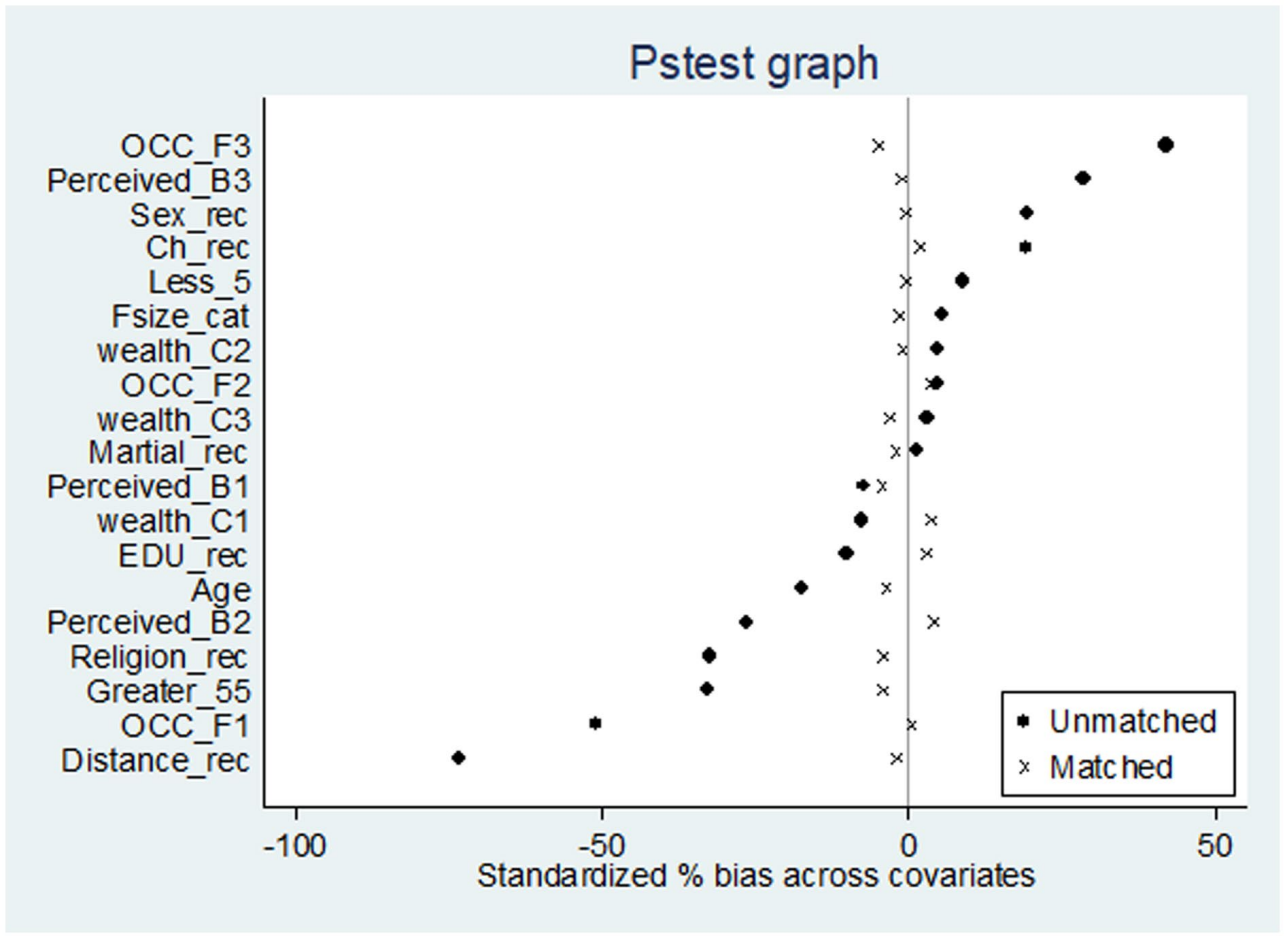
Pstest graph of samples, both matched and unmatched.

These variables fulfilled the PSM model conditional independence assumption were used in probit regression to estimate the propensity score (Pscore). A kernel matching algorithm with a bandwidth of 0.06 was selected after balancing was checked using Pstest. Then estimation of the effect of CBHI on health service utilization was done using kernel matching (with bandwidth 0.06). The comparison of density estimation of both treated and untreated groups before matching and after matching was done (Table 4). Beyond numeric balance, we also assessed covariate balance visually with a standard difference plot (Figure 3).

**Table 4:** Pstest estimation result.

Variable	Unmatched matched	Mean		% bias	% reduct│bias│	*t*-test		V(T)/V(C)
		Treated	Control			T	*p* > |*t*|	
Age	U	44.61	47.01	−17.2	76.70	−2.23	0.03	0.51*
	M	44.61	45.19	−4.00		−0.53	0.59	0.60
Family size	U	0.53	0.50	5.70	71.90	0.74	0.46	.
	M	0.52	0.53	−1.60		−0.20	0.84	.
Marital status	U	0.68	0.68	1.40	−39.10	0.18	0.86	.
	M	0.68	0.69	−1.90		−0.24	0.81	.
Sex	U	0.63	0.54	19.50	97.80	2.53	0.01	.
	M	0.62	0.62	−0.40		−0.05	0.96	.
Religion	U	0.92	0.99	−32.50	87.20	−4.23	0.00	.
	M	0.96	0.97	−4.20		−0.57	0.57	.
Farmer	U	0.09	0.29	−50.90	98.90	−6.59	0.00	.
	M	0.11	0.10	0.60		0.09	0.93	.
Labor work	U	0.59	0.56	4.90	31.50	0.64	0.52	.
	M	0.66	0.61	3.40		0.43	0.67	.
Merchant	U	0.31	0.14	42.00	89.10	5.46	0.00	.
	M	0.28	0.29	−4.60		−0.52	0.60	.
Poor	U	0.40	0.44	−7.40	45.60	−0.95	0.34	.
	M	0.42	0.40	4.00		0.50	0.62	.
Middle	U	0.27	0.25	5.00	69.50	0.64	0.52	.
	M	0.26	0.27	−1.50		−0.19	0.85	.
Rich	U	0.33	0.31	3.10	9.30	0.40	0.69	.
	M	0.32	0.33	−2.80		−0.35	0.73	.
Educational status	U	0.31	0.35	−10.01	67.30	−1.31	0.19	.
	M	0.30	0.29	3.30		0.43	0.67	.
Poor perception toward family health	U	0.08	0.11	−7.20	41.20	−0.94	0.35	.
	M	0.09	0.10	−4.30		−0.53	0.59	.
Medium perception toward family health	U	0.20	0.32	−26.30	83.90	−3.41	0.00	.
	M	0.21	0.19	4.20		0.58	0.56	.
Good perception toward family health	U	0.71	0.57	28.60	95.60	3.71	0.00	.
	M	0.70	0.71	−1.30		−0.16	0.87	.
Distance to health facility	U	0.02	0.26	−73.30	97.70	−9.46	0.00	.
	M	0.02	0.03	−1.70		−0.45	0.65	.
Children younger than 5 in the family	U	0.56	0.52	8.90	97.10	1.15	0.25	.
	M	0.56	0.56	−0.30		−0.03	0.97	.
Member older than 55 in the family	U	0.28	0.43	−32.60	87.40	−4.22	0.00	.
	M	0.29	0.31	−4.10		−0.53	0.59	.
Presence of chronic illness	U	0.27	0.18	19.30	88.10	2.50	0.01	.
	M	0.27	0.26	2.30		0.27	0.79	
Illness in the past one year in the family	U	0.99	0.10	−4.60	89.80	−0.60	0.55	
	M	1.00	0.99	0.50		0.31	0.75	

*If variance ratio outside [0.81; 1.24] for U and [0.80; 1.25] for M.

This study indicated that enrolling in the CBHI program has a significant impact on health service utilization. Hence, CBHI enrolled households have a 6.9 percentage points (ATT = 0.069; 95% CI: 0.034, 0.114) increase in health service utilization than non-enrolled household groups (Table 5).

**Table 5:** Average treatment effect of CBHI on health service utilization in Dabat and Woreta town northwest Ethiopia, 2021 (*n* = 644).

Treated	Comparison	ATT	SE	T-test	95% CI
332	312	0.069	0.021	3.316	0.033619, 0.114382

ATT, Average treatment effect on treated; SE, Standard Error; CI, Confidence Interval.

### Checking robustness of ATT

Robustness was checked to increase the reliability of the result, which is described in Table 6. The four matching algorithms were checked to get the impact of CBHI on health service utilization and similar results were found from each matching algorithm to show the robustness of the findings.

**Table 6:** Checking robustness of ATT.

Matching algorithm	Treated (CBHI enrolled)	Comparison (non-enrolled)	ATT	SE	T-test
Nearest Neighbor matching (NNM)					
NNM (0.01)	332	132	0.090	0.030	2.992
NNM (0.01)	332	132	0.090	0.030	3.055
NNM without replacement	332	132	0.090	0.033	2.747
Radius matching (R)					
R (0.01)	300	283	0.080	0.026	3.077
R (0.25)	332	312	0.078	0.020	3.943
R (0.5)	332	312	0.112	0.023	4.968
Kernel matching (K)					
K (0.01)	332	312	0.069	0.022	3.149
K (0.06)	332	312	0.069	0.021	3.316
K (0.25)	332	312	0.069	0.019	3.682
K (0.5)	332	312	0.069	0.022	3.094
Stratification	332	312	0.070	0.018	3.814

ATT, Average treatment effect on treated; SE, Standard Error.

In summary, the weight was given for the dimension of outcome evaluation by effectiveness and impact, and the overall program score was 75.2%. Thus, the program achieves its objective in a satisfactory condition, which is presented in Table 7.

**Table 7:** Summary of the overall performance of CBHI program in Dabat and Woreta town, northwest Ethiopia, 2021.

Dimensions	Weight	Score	Achievement	Judgment
Effectiveness	50	40.62	81.3	Very Good
Impact	50	34.5	69	Fair
Overall outcome of CBHI			75.2	***Satisfactory***

Score is calculated: (Observed*weight)/expected; Achievement is calculated: (Score/weight) * 100%.

### Challenges of CBHI program effectiveness

In this qualitative method, a thematic analysis was done, and a theme generated from all the in-depth and key informant interviews (KII) which focused on the challenges to visit health facilities from the services user’s perspectives and the difficulties that were faced during service provision. Accordingly, four themes were identified: (i) availability of health professionals in CBHI contracted health facilities, (ii) availability of essential drugs and medical supplies, (iii) longer waiting times, and (iv) claims to reimbursement of health expenditure.

#### Availability of health professionals in CBHI-contracted health facilities

Key informant interviewees acknowledged that the number of health professionals remains inadequate despite recent recruitment into CBHI-contracted health centers.

*“Our health center is the only one in town, and it serves a large population. As a result, the numbers of health professionals are not matched to the patients who visit the facility; this is a major problem that we encountered while providing service to clients. We attempted to inform relevant stakeholders in order to solve this problem; however, the matter remains unsolved despite the recent recruitment of two doctors”* (42 years KI’s).

This idea was supported by the key informants’ findings.

*“The number of patients at the health center grew over time; however, the number of health workers wasn't sufficient to handle the increasing number of patients. Due to this, the client does not get the required service as quickly as they would like. We tried to communicate with the higher officials to come up with a solution for this problem”* (32 years, KIs).

#### Availability of essential drugs and medical supplies

Most CBHI members complained that there are shortages of essential drugs and medical supplies in the health facilities.

*“Most of the time, pharmacists told us drugs are not available and we often must buy from private retailers outside the public facilities. Paying out of pocket is a problem for us because we have already paid a registration fee and premium and have no money for these services. For this reason, we borrowed from others to buy the drug and the program did not reimburse the money within a short time. As a result, my family and I did not want to visit the health facility unless the illness was life threatening”*(46 years, CBHI member).

The finding is also supported from the KII.

“*In general, the main problems that we faced in our health center are lack of resources (drug and medical equipment) and high workload on health workers*” (42 years KI’s).

#### Long waiting times

The participants reported that the amount of time they waited to be seen by health professionals affected their service utilization at health facilities.

*“We spent a long time waiting to get service; sometimes it may take more than a day. Due to this we suffered much in addition to our illness. The possible cause, in my opinion, is that the number of patients is not balanced with the number of health professionals. This makes my family and me hesitant to visit the health center”* (58 years, CBHI member).

This finding is also confirmed from another CBHI member in-depth interview.

*“Since yesterday, I've been here for service, and after spending a full day here, the health care provider scheduled me for today because of the high number of patients. We are suffering a lot to get service, and we spent a day and more to get service. As a result, our illness became complicated, but it could be easily treated if the waiting time is shortened”* (36 years, CBHI member).

#### Claims to reimbursement of health expenditure

The program enters into contract agreements with health centers to provide services per the benefits package for CBHI beneficiaries and to reimburse them; however, the program’s financial sources find it difficult to reimburse the health facilities.

*“Currently, the program does not provide reimbursement to CBHI-contracted health centers timely. This is a difficult situation for our health center, as well as other health facilities. If the health expenditure (money) is not reimbursed at the time, our budget does not allow us to purchase the resources that are required for service. As a result, we are experiencing troubles in accessing medications and other vital medical supplies, and the quality of our health-care facilities will remain static if the program continues in this manner”* (42 years, KI’s).

This idea was confirmed by the key informants’ findings; there were no sufficient resources to reimburse the health expenditure for the CBHI-contracted health facilities.

“*The program has trouble in reimbursing the CBHI-contracted health facility's health expenses. Last year, we earned 1.3 million Ethiopian birrs from the annual membership payment. However, the overall cost of health services used by members was roughly 3 million Ethiopian birrs. The program was confronted with difficulties as a result of this significant disparity”* (32 years KII’s).

*“Our health center did not obtain the reimburse birr for service that the members rendered at the time. In my opinion, to solve this problem the program should review premium payment and consider some escalation. This helps the program to have an adequate financial resource besides the government subsidies. Then the health facility gets a chance to improve the health service quality (by improving the availability of essential drugs and medical equipment’s including laboratory service)”* (42 years KI’s).

## Discussion

In this evaluation, the OECD criteria were used to estimate the effectiveness and impact of the CBHI program on health service utilization. The findings showed a utilization rate of 1.3 visits per individual per year of health service among CBHI members, and according to the judgment criteria the effectiveness and impact of CBHI was 81.3 and 69%, respectively. The overall outcome of CBHI was judged as 75.2%, which is satisfactory.

In our evaluation, the health service utilization rate among CBHI members (1.3 visits per individual per year) is below the program’s target objective (1.6 visits per individual per year). The CBHI program is known to provide financial protection by reducing OOP expenditure and increase access to inpatient and outpatient services (14). However, this evaluation revealed that there are challenges that prevent the CBHI program from progressing toward achieving health service utilization as intended, including an inadequate number of health professionals to handle the increasing number of clients in the health facility. This means clients spend a long time waiting to get service; sometimes it may take more than a day because the number of clients and health professionals is not balanced, and this makes them hesitant to visit the health facilities. In addition, health professionals faced high workloads without additional payments, which made them less motivated to provide care or health services for their clients. Clients became reluctant to visit the health facilities and avoided treatment or care due to the less motivated and fatigued health professionals. In addition, non-reimbursement of health facilities’ expenditure by the CBHI office and out-of-stock drugs and medical supplies were identified as challenges. This implies that the CBHI program should develop strategies to review premium payments and make some modifications. This would help the program to have an adequate financial resource besides the government subsidies to reimburse the health facility expenditure. As a result, health facilities would get a chance to improve the quality of service (by improving the availability of essential drugs and medical equipment, including laboratory services and adequate numbers of human resources). Consequently, the CBHI program can progress toward achieving health service utilization as the stated objective.

A similar study conducted in India showed that one of the biggest challenges faced by the CBHI program is related to the reimbursement of medical costs of CBHI members for health facilities; as a result, attaining a good balance between serving the poor and maintaining financial sustainability is difficult (6). This could be for a reason for the poor quality of CBH- contracted health facilities. Another study showed that the CBHI insurance contract does not show any difference in health service utilization among CBHI enrolled and non-enrolled individuals (69). A possible explanation for this could be the capitation system (paying a fixed fee per patient per year) (70), poor health service quality, and members being faced with difficulty paying bills if the CBHI does not reimburse health expenditures after they have paid for the service (71). Another possible explanation could be the type of service assessed in the study; the program might not have an effect on exempted services such as maternal service (72).

In this evaluation, we found that the average treatment effect of CBHI on health service utilization is 6.9 percentage points (ATT = 0.069; 95% CI: 0.034, 0.114), which showed a significant impact of CBHI on health service utilization. Hence, CBHI-enrolled households have health service utilization rates that are 6.9 percentage points higher per individual per year than households that are not enrolled. Our findings are higher than those in a similar study in Vietnam (73); this difference could be attributable to the fact that our study included both outpatient and inpatient services. However, this finding was lower than a previous study conducted in Ethiopia (42) and Rwanda (12). The observed discrepancy could be due to the differences in health service utilization measurement. In these studies, the health service utilization status of CBHI members was measured after illness episodes, whereas in our study all members were assessed, regardless of their illness history.

### Strength and limitations of the evaluation

Our results provided compelling evidence on whether the CBHI program is effective or not in achieving its objective and also its effect on health service utilization using rigorous methodology. As a result, we assessed the outcome difference between the intervention and comparison groups by matching the baseline characteristics. In addition, possible challenges for not achieving the program objective as planned were explained qualitatively.

However, there are some limitations, so further work should be done on overall outcome evaluation, including all outcome level changes, i.e., health-seeking behavior, health facility quality, and OOP health expenditure, in order to make an overall decision on the effectiveness of the program. The evaluation is limited due to selection bias; randomization was not feasible in this program evaluation (the piloted/intervention group was already started) study, so the results may be overestimated or underestimated. But use of a quasi-experimental evaluation design, specifically a matched comparison group, is recommended to overcome the randomization issue. The PSM model was also employed to minimize the selection bias while estimating the average treatment effect. In addition, the participants might have experienced recall bias, particularly regarding the services they experienced in the last 12 months before the evaluation. To overcome this bias, the data collectors were highly experienced and well-trained on the tools to explain the questions and respondents were allowed to recall events later. On top of that, mixed method was employed and careful selection was made of evaluation questions. According to some recommendations, in a district, the whole kebele must be considered if their numbers are less than nine. However, in our study, taking the whole kebele was not feasible because of the nature of the program implementation (CBHI was piloted in Woreta) and also other factors such as the data collection time and resources. Moreover, our evaluation nature is goal-oriented, which means a type of evaluation that seeks to determine if the stated goals and objectives of the program or project have been achieved or not. As a result, we could not observe and measure all actual outcomes and judge the program according to its positive and negative effects on its members due to financial problems. Therefore, in the future, we recommend researchers consider a goal-free evaluation to capture the negative effect of the CBHI program on clients. Lastly, baseline data for both the intervention and comparison groups were not available. In that regard, we use the post-test only comparison design, but it might be better if the pre-post comparison design is applied to estimate the true effect of CBHI effectiveness and its impact on health service utilization.

## Conclusion

The evaluation showed that the overall outcome of CBHI toward health service utilization was judged as satisfactory. The CBHI program showed an improvement in health service utilization among CBHI members. However, the health service utilization rate among CBHI members is less than the target stated for the program and the WHO’s recommendation.

As a result, measures should be taken to address the identified challenges of program implementation toward health service utilization, i.e., inadequate number of health professionals, longer waiting times to get service, health facility expenditure not reimbursable by the CBHI office, and shortage of drugs and medical supplies. Hence, appropriate and timely reimbursement of resources is essential to increase absolute resources and improve service quality.

### Sample interview outline

1. Even though the effectiveness of the program is good, the health service utilization rate is relatively lower as compared to program objectives: what do you think the reasons for this could be? (Quality of health service improved, paying the premium at the time, renewing membership at the time, avoiding moral hazards, financial coverage for members).
2. What challenges has the program faced during implementation?
3. Did the program have any solutions for identified challenges?

## Data availability statement

The original contributions presented in the study are included in the article/supplementary material, further inquiries can be directed to the corresponding author.

## Ethics statement

The studies involving humans were approved by Ethical committee of the Institute of Public Health, College of Medicine and Health Sciences, University of Gondar (Ref No: IPH/1461/2013). The studies were conducted in accordance with the local legislation and institutional requirements. The participants provided their written informed consent to participate in this study.

## Author contributions

All authors contributed to the preparation of the manuscript. SF conceived and designed the idea and performed the analysis. MM and AA were advisors in the proposal and thesis writing, and revised the final drafts of the manuscript. SF did the data extraction, analysis, interpretation of the finding, and writing the manuscript. All authors contributed to the article and approved the submitted version.

## References

[1] World Health Organization. The world health report: Health systems financing: The path to universal coverage. Geneva: World Health Organization (2010). 10.2471/BLT.10.078741 PMC2878164 20539847

[2] Shahrawat RR Rao KD. Insured yet vulnerable: out-of-pocket payments and India's poor. Health Policy Plan. (2012) 27:213–21. doi: 10.1093/heapol/czr029, PMID: 21486910

[3] Van Minh H Phuong NTK Saksena P James CD Xu K. Financial burden of household out-of pocket health expenditure in Viet Nam: findings from the National Living Standard Survey 2002–2010. Soc Sci Med. (2013) 96:258–63. doi: 10.1016/j.socscimed.2012.11.028, PMID: 23246399

[4] Van Doorslaer E O'Donnell O Rannan-Eliya RP Somanathan A Adhikari SR Garg CC. Catastrophic payments for health care in Asia. Health Econ. (2007) 16:1159–84. doi: 10.1002/hec.1209 17311356

[5] McIntyre D Thiede M Dahlgren G Whitehead M. What are the economic consequences for households of illness and of paying for health care in low-and middle-income country contexts? Soc Sci Med. (2006) 62:858–65. doi: 10.1016/j.socscimed.2005.07.001, PMID: 16099574

[6] Purohit B. Community based health Insurance in India: prospects and challenges. Health. (2014) 6:1237–45. doi: 10.4236/health.2014.611152

[7] Agency EHI. Evaluation of community-based health insurance pilot schemes in Ethiopia. Oxford: Oxford University Press in association with the London School of Hygiene and Tropical Medicine (2015).

[8] Oriakhi H Onemolease E. Determinants of rural household’s willingness to participate in community based health insurance scheme in Edo state. Nigeria Stud ethno-medicine. (2012) 6:95–102. doi: 10.1080/09735070.2012.11886425

[9] Mekonen AM Gebregziabher MG Teferra AS. The effect of community based health insurance on catastrophic health expenditure in Northeast Ethiopia: a cross sectional study. PloS One. (2018) 13:e0205972. doi: 10.1371/journal.pone.0205972, PMID: 30335838 PMC6193712

[10] Jütting JP. Do community-based health insurance schemes improve poor people’s access to health care? Evidence Rural Senegal World Develop. (2004) 32:273–88. doi: 10.1016/j.worlddev.2003.10.001

[11] Yilma Z Mebratie A Sparrow R Dekker M Alemu G Bedi AS. Impact of Ethiopia's community based health insurance on household economic welfare. World Bank Econ Rev. (2015) 29:S164–73. doi: 10.1093/wber/lhv009

[12] Shimeles A. Community based health insurance schemes in Africa: The case of Rwanda. (2010). Working Papers in Economics 463. Available at: http://hdl.handle.net/2077/23064 (Accessed October 30, 2023).

[13] Gnawali DP Pokhrel S Sié A Sanon M De Allegri M Souares A. The effect of community-based health insurance on the utilization of modern health care services: evidence from Burkina Faso. Health Policy. (2009) 90:214–22. doi: 10.1016/j.healthpol.2008.09.015, PMID: 19036467

[14] Ekman B. Community-based health insurance in low-income countries: a systematic review of the evidence. Health Policy Plan. (2004) 19:249–70. doi: 10.1093/heapol/czh031, PMID: 15310661

[15] Aggarwal A. Impact evaluation of India's ‘Yeshasvini’community-based health insurance programme. Health Econ. (2010) 19:5–35. doi: 10.1002/hec.1605, PMID: 20803629

[16] Mebratie A Sparrow R Debebe ZY Abebaw D Alemu G Bedi AS. The impact of Ethiopia’s pilot community based health insurance scheme on healthcare utilization and cost of care. ISS Working Paper Series/General Series. (2014) 593:1–46. 10.1016/j.socscimed.2018.11.003 30419495

[17] Ahmed S Sarker AR Sultana M Chakrovorty S Ahmed MW Dorin F. The impact of community-based health insurance on the utilization of medically trained healthcare providers among informal workers in Bangladesh. PloS One. (2018) 13:e0200265. doi: 10.1371/journal.pone.0200265, PMID: 29995899 PMC6040718

[18] Nshakira-Rukundo E Mussa EC Nshakira N Gerber N von Braun J. Impact of community-based health insurance on utilisation of preventive health services in rural Uganda: a propensity score matching approach. Int J Heal Econ Manag. (2021) 21:203–27. doi: 10.1007/s10754-021-09294-6, PMID: 33566252 PMC8192361

[19] Haven N Dobson AE Yusuf K Kellermann S Mutahunga B Stewart AG. Community-based health insurance increased health care utilization and reduced mortality in children under-5, around Bwindi community hospital, Uganda between 2015 and 2017. Front Public Health. (2018) 6:281. doi: 10.3389/fpubh.2018.00281, PMID: 30356909 PMC6190927

[20] Smith KV Sulzbach S. Community-based health insurance and access to maternal health services: evidence from three west African countries. Soc Sci Med. (2008) 66:2460–73. doi: 10.1016/j.socscimed.2008.01.044, PMID: 18362047

[21] Wang W Temsah G Mallick L. The impact of health insurance on maternal health care utilization: evidence from Ghana, Indonesia and Rwanda. Health Policy Plan. (2017) 32:366–75. doi: 10.1093/heapol/czw135, PMID: 28365754 PMC5400062

[22] Nageso D Tefera K Gutema K. Enrollment in community based health insurance program and the associated factors among households in Boricha district, Sidama zone, southern Ethiopia; a cross-sectional study. PloS One. (2020) 15:e0234028. doi: 10.1371/journal.pone.0234028, PMID: 32484840 PMC7266314

[23] Abdilwohab MG Abebo ZH Godana W Ajema D Yihune M Hassen H. Factors affecting enrollment status of households for community based health insurance in a resource-limited peripheral area in southern Ethiopia. Mixed method PloS one. (2021) 16:e0245952. doi: 10.1371/journal.pone.0245952, PMID: 33493240 PMC7833211

[24] Mebratie AD Sparrow R Yilma Z Alemu G Bedi AS. Enrollment in Ethiopia’s community-based health insurance scheme. World Dev. (2015) 74:58–76. doi: 10.1016/j.worlddev.2015.04.011 25616670

[25] Derseh A Sparrow R Debebe ZY Alemu G Bedi AS. Enrolment in Ethiopia’s community based health insurance scheme. ISS Working Paper Series/General Series. (2013) 578:1–35.

[26] Ashagrie B Biks GA Belew AK. Community-based health insurance membership dropout rate and associated factors in Dera District, Northwest Ethiopia. Risk Manag Healthcare Policy. (2020) 13:2835–44. doi: 10.2147/RMHP.S277804, PMID: 33304111 PMC7723227

[27] Mebratie AD Sparrow R Yilma Z Alemu G Bedi AS. Dropping out of Ethiopia’s community-based health insurance scheme. Health Policy Plan. (2015) 30:1296–306. doi: 10.1093/heapol/czu142, PMID: 25616670

[28] Eseta WA Lemma TD Geta ET. Magnitude and determinants of dropout from community-based health insurance among households in manna district, Jimma zone, Southwest Ethiopia. Clinicoecon Outcomes Res: CEOR. (2020) 12:747–60. doi: 10.2147/CEOR.S284702, PMID: 33364800 PMC7751608

[29] Terfasa TG. Community-based health Insurance coverage, dropout rate and factors associated with among households in selected districts of west Shoa zone, Ethiopia 2017/18. In: 30th EPHA Annual Conference: 2019. (2019).

[30] Kwon S. Community-based health Insurance in Ethiopia: Enrollment, memebrship renewal, and effects on health service utilization 서울대학교 대학원 (2018).

[31] Kaso AW Yohanis Y Debela BG Hareru HE. Community-based health Insurance membership renewal rate and associated factors among households in Gedeo zone, southern Ethiopia. J Environ Public Health. (2022) 2022:1–11. doi: 10.1155/2022/8479834, PMID: 36225760 PMC9550414

[32] Badacho AS Tushune K Ejigu Y Berheto TM. Household satisfaction with a community-based health insurance scheme in Ethiopia. BMC Res Notes. (2016) 9:1–10. doi: 10.1186/s13104-016-2226-9 27576468 PMC5004312

[33] Hailie MT Hassen SL Temesgen MM. Client satisfaction on community based health insurance scheme and associated factors at Boru Meda hospital, northeast, Ethiopia: institutional based cross-sectional study. BMC Health Serv Res. (2021) 21:1–8. doi: 10.1186/s12913-021-07223-4 34847939 PMC8630846

[34] Mitiku Kebede K Geberetsadik SM. Household satisfaction with community-based health insurance scheme and associated factors in piloted Sheko district; Southwest Ethiopia. PloS One. (2019) 14:e0216411. doi: 10.1371/journal.pone.0216411, PMID: 31083706 PMC6513074

[35] Agency EHI. Evaluation of community-based health insurance pilot schemes in Ethiopia. Ethiopia: Ethiopian health insurance agency Addis Ababa (2015).

[36] Mebratie AD Sparrow R Yilma Z Abebaw D Alemu G Bedi AS. The impact of Ethiopia's pilot community based health insurance scheme on healthcare utilization and cost of care. Soc Sci Med. (2019) 220:112–9. doi: 10.1016/j.socscimed.2018.11.003, PMID: 30419495

[37] Dagnaw FT Azanaw MM Adamu A Ashagrie T Mohammed AA Dawid HY. Community-based health insurance, healthcare service utilization and associated factors in South Gondar zone northwest, Ethiopia, 2021: a comparative cross-sectional study. PloS One. (2022) 17:e0270758. doi: 10.1371/journal.pone.0270758, PMID: 35789337 PMC9255736

[38] Atnafu DD Tilahun H Alemu YM. Community-based health insurance and healthcare service utilisation, north-west, Ethiopia: a comparative, cross-sectional study. BMJ Open. (2018) 8:e019613. doi: 10.1136/bmjopen-2017-019613, PMID: 30093509 PMC6089309

[39] Jembere M. Community based health insurance scheme as a new healthcare financing approach in rural Ethiopia: role on access, use and quality of healthcare services, the case of tehuledere district, south wollo zone, Northeast Ethiopia. Family Med Med Sci Res. (2018) 7:1–3. doi: 10.4172/2327-4972.1000227

[40] Fite MB Roba KT Merga BT Tefera BN Beha GA Gurmessa TT. Factors associated with enrollment for community-based health insurance scheme in Western Ethiopia: case-control study. PloS One. (2021) 16:e0252303. doi: 10.1371/journal.pone.0252303, PMID: 34111135 PMC8191870

[41] Demissie B Negeri KG. Effect of community-based health insurance on utilization of outpatient health care services in southern Ethiopia: a comparative cross-sectional study. Risk Manag Healthcare Policy. (2020) 13:141–53. doi: 10.2147/RMHP.S215836, PMID: 32158291 PMC7049267

[42] Mebratie AD Sparrow R Yilma Z Abebaw D Alemu G Bedi A. Impact of Ethiopian pilot community-based health insurance scheme on health-care utilisation: a household panel data analysis. Lancet. (2013) 381:S92. doi: 10.1016/S0140-6736(13)61346-X

[43] Kassie G Tefera B. Effects of community-based health insurance on modern family planning utilization in Ethiopia. Gates Open Res. (2019) 3:1461. doi: 10.12688/gatesopenres.12960.2

[44] Atnafu A Gebremedhin T. Community-based health Insurance Enrollment and child health service utilization in Northwest Ethiopia: a cross-sectional case comparison study. Clinicoecon Outcomes Res: CEOR. (2020) 12:435–44. doi: 10.2147/CEOR.S262225, PMID: 32848434 PMC7428314

[45] Alemayehu YK Dessie E Medhin G Birhanu N Hotchkiss DR Teklu AM. The impact of community-based health insurance on health service utilization and financial risk protection in Ethiopia. BMC Health Serv. Res. (2023) 23:67 10.1186/s12913-022-09019-6 PMC9869550 36683041

[46] Trujillo AJ Portillo JE Vernon JA. The impact of subsidized health insurance for the poor: evaluating the Colombian experience using propensity score matching. Int J Health Care Finance Econ. (2005) 5:211–39. doi: 10.1007/s10754-005-1792-5, PMID: 16082516

[47] Wagstaff A. Estimating health insurance impacts under unobserved heterogeneity: the case of Vietnam's health care fund for the poor. Health Econ. (2010) 19:189–208. doi: 10.1002/hec.1466, PMID: 19248053

[48] Mensah J Oppong JR Schmidt CM. Ghana's National Health Insurance Scheme in the context of the health MDGs: an empirical evaluation using propensity score matching. Health Econ. (2010) 19:95–106. doi: 10.1002/hec.1633, PMID: 20730999

[49] Shigute Z Mebratie AD Sparrow R Alemu G Bedi AS. The effect of Ethiopia’s community-based health insurance scheme on revenues and quality of care. Int J Environ Res Public Health. (2020) 17:8558. doi: 10.3390/ijerph17228558, PMID: 33218111 PMC7698817

[50] Chianca T. The OECD/DAC criteria for international development evaluations: an assessment and ideas for improvement. J Multidis Evalu. (2008) 5:41–51. doi: 10.56645/jmde.v5i9.167

[51] Mebratie AD. Essays on evaluating a community based health insurance scheme in rural Ethiopia [Internet]. [ISS PhD Theses]. International Institute of Social Studies of Erasmus University (ISS). (2015) Available at: http://hdl.handle.net/1765/78363

[52] Ministry of Health: Essential Health Service Packages of Ethiopia. (2019) Available at: https://www.humanitarianresponse.info/sites/www.humanitarianresponse.info/files/documents/files/essential_health_services_package_of_ethiopia_2019.pdf (Accessed October 30, 2023).

[53] Ministry of Health: HMIS Indicator Reference Guide Technical Standards: Area 1, (2017) Available at: http://repository.iifphc.org/handle/123456789/392

[54] Ethiopia National Health Insurance: CBHI program annual plan (2021).

[55] Hanita M Ansel D Shakman K. Matched-comparison group design: An evaluation brief for educational stakeholders. Education development Center. (2017) Available at: https://oese ed gov/files/2019/03/02-09-MatchedComparison pdf 2017.

[56] Csa I. Central statistical agency (CSA)[Ethiopia] and ICF. Addis Ababa, Ethiopia and Calverton, Maryland, USA: Ethiopia demographic and health survey (2016).

[57] Adebayo EF Uthman OA Wiysonge CS Stern EA Lamont KT Ataguba JE. A systematic review of factors that affect uptake of community-based health insurance in low-income and middle-income countries. BMC Health Serv Res. (2015) 15:1–13. doi: 10.1186/s12913-015-1179-3 26645355 PMC4673712

[58] Bennett S. The role of community-based health insurance within the health care financing system: a framework for analysis. Health Policy Plan. (2004) 19:147–58. doi: 10.1093/heapol/czh018, PMID: 15070863

[59] Birmeta K Sim B Kim D Dhakal S Do Y. Analyzing barriers to accessing health care services in Holeta town. Ethiopia Prim Health Care. (2015) 5:1–7. doi: 10.4172/2167-1079.1000204

[60] Anney VN. Ensuring the quality of the findings of qualitative research: looking at trustworthiness criteria. J Emerg Trends Educ Res Policy Stud. (2014) 5:272–81.

[61] Yao XI Wang X Speicher PJ Hwang ES Cheng P Harpole DH. Reporting and guidelines in propensity score analysis: a systematic review of cancer and cancer surgical studies. JNCI: J National Cancer Institute. (2017) 109:djw323:7–9 doi: 10.1093/jnci/djw323 PMC6059208 28376195

[62] Shadish WR Steiner PM. A primer on propensity score analysis. Newborn Infant Nurs Rev. (2010) 10:19–26. doi: 10.1053/j.nainr.2009.12.010

[63] Pearl J. On a class of bias-amplifying variables that endanger effect estimates. arXiv preprint arXiv:12033503. (2012).

[64] Pearl J. Causal inference in statistics: an overview. Stat surveys. (2009) 3:96–146. doi: 10.1214/09-SS057

[65] Steiner PM Cook TD Shadish WR. On the importance of reliable covariate measurement in selection bias adjustments using propensity scores. J Educ Behav Stat. (2011) 36:213–36. doi: 10.3102/1076998610375835

[66] Zhao Z. Data issues of using matching methods to estimate treatment effects: an illustration with NSW data set. China Center for Econ Res. (2003) 138:1–37.

[67] Rosenbaum PR Rubin DB. Constructing a control group using multivariate matched sampling methods that incorporate the propensity score. Am Stat. (1985) 39:33–8. doi: 10.1080/00031305.1985.10479383

[68] Stöckl H Filippi V Watts C Mbwambo JK. Induced abortion, pregnancy loss and intimate partner violence in Tanzania: a population based study. BMC Pregnancy Childbirth. (2012) 12:12. doi: 10.1186/1471-2393-12-12, PMID: 22390254 PMC3311557

[69] Raza WA Van de Poel E Bedi A Rutten F. Impact of community-based health insurance on access and financial protection: evidence from three randomized control trials in rural India. Health Econ. (2016) 25:675–87. doi: 10.1002/hec.3307, PMID: 26708298

[70] Fink G Robyn PJ Sié A Sauerborn R. Does health insurance improve health?: evidence from a randomized community-based insurance rollout in rural Burkina Faso. J Health Econ. (2013) 32:1043–56. doi: 10.1016/j.jhealeco.2013.08.003 24103498

[71] Umeh CA Feeley FG. Inequitable access to health care by the poor in community-based health insurance programs: a review of studies from low-and middle-income countries. Global Heal Sci Prac. (2017) 5:299–314. doi: 10.9745/GHSP-D-16-00286, PMID: 28655804 PMC5487091

[72] Mills A. Health policy and systems research: defining the terrain; identifying the methods. Health Policy Plan. (2012) 27:1–7. doi: 10.1093/heapol/czr006, PMID: 21324972

[73] Thuong NTT. Impact of health insurance on healthcare utilisation patterns in Vietnam: a survey-based analysis with propensity score matching method. BMJ Open. (2020) 10:e040062. doi: 10.1136/bmjopen-2020-040062, PMID: 33046477 PMC7552874

